# A review of educational-based gambling prevention programs for adolescents

**DOI:** 10.1186/s40405-017-0024-5

**Published:** 2017-06-07

**Authors:** Boon Chin Oh, Yee Jie Ong, Jasmine M. Y. Loo

**Affiliations:** 1 Graduate School of Education, University of Melbourne, Parkville, VIC 3010 Australia; 2 Psychology Department, Anglia Ruskin University, East Rd, Cambridge, UK; 3 Jeffrey Cheah School of Medicine and Health Sciences, Monash University Malaysia, Sunway, Selangor DarulEhsan Malaysia

## Abstract

Educational-based problem gambling prevention programs are important avenues in targeting at-risk behaviors among adolescents to prevent an escalation of problematic behaviors into adulthood. The aim of this review is to examine features pertinent to effective educational-based programs in the area of adolescent problem gambling prevention in hopes of providing a foundation and future suggestions for preventive efforts. A stronger understanding of this research area will be essential in ensuring that past practical and theoretical advancements are integrated into the development of future programs.

## Background

Gambling disorder (GD) is defined as a continuous and recurring compulsive gambling behavior that may affect functioning at the individual, family and social level (American Psychiatric Association 2013; Pirritano et al. 2014; Verdura Vizcaino et al. 2013). It is formally known as pathological gambling (PAG) in DSM-IV, which is defined as destructive and recurrence gambling behavior that intervene interest regarding personal, family and career (Loo et al. 2008; Sleczka et al. 2015). It is used primarily in clinical and medical contexts. Meanwhile, problem gambling (PG) is defined as compulsive gambling behavior that bring forth negative consequences to personal and society, but it may not necessarily meet diagnostic criteria of pathological gambling (Loo et al. 2008; Williams et al. 2012). Hence, these terms (GD, PAG, PG) will guide our discussion in this paper.

Nowadays, gambling can be easily accessed in many forms (Lavoie and Ladouceur 2004). Given the global expansion of the gambling industry, a significant increase in the prevalence of problem gambling is inevitable (Williams et al. 2012). Williams et al. (2012) compared various problem gambling prevalence studies from 1975 to 2012 and identified an average PG rate of 2.3% across the different countries, with the lowest 0.5% in Denmark and Netherland, and the highest in Hong Kong (average 5.6%), Macau (6.0%) and South Africa (6.4%). Lower PG rates were found in European countries (e.g., Denmark and Germany), while higher rates were found in Asian countries (e.g. Hong Kong, Macau, and Singapore) (Williams et al. 2012).

This globalise phenomenon is associated with numerous negative consequences, affecting not just individuals, but as well as their families and the society (Messerlian and Derevensky 2005; Williams et al. 2012). Gambling industries were reported to be generating remarkable profits with over US$ 30 billion revenues from nations such as United States and Macau (Gaming Inspection and Coordination Bureau 2012; Lehman 2011). With such a high involvement of money on gambling, there is no doubt that financial difficulties would be one of the most common problem experienced as PGs have been reported suffering from massive debts, poverty and bankruptcy (George and Murali 2005; Williams et al. 2011). This can lead to other secondary issues such as disruption in family relationships. PGs would often have conflicts with their family members, potentially negatively impacting younger generations. Adolescents who report symptoms of GD are more likely to have parents with gambling issues (Magoon and Ingersoll 2006; Vachon et al. 2004). Similarly with adults, adolescents also face many psychosocial issues associated with PG.

Problem gamblers are also at a higher risk of developing many psychological issues, namely, depression, anxiety, alcoholism and antisocial personality disorder (Cunningham-Williams et al. 1998; Delfabbro et al. 2006; George and Murali 2005). Among them, depression is most prevalent, with a comorbidity range of 50–70%, and this is prominent among youths (Becona et al. 1996; Messerlian et al. 2007). Langhinrichsen-Rohling et al. (2004) also found that problematic adolescent gamblers were more susceptible to conduct problems, substance abuse and emotional issues. Relational issues may consequently lead to family neglect, domestic violence and even mental health problems (George and Murali 2005; Williams et al. 2012). In fact, it was reported that children of problem gamblers were at a higher risk of developing conduct and adjustment problems, mostly due to neglected parenting precipitated by gambling activities (Vitaro et al. 2008). There is no doubt that an adolescent’s school performance would also be affected as their attention is being redirected to managing gambling-related problems. Ólason et al. (2006) reported that students who engage in PG reported lower grades, lower satisfaction of their school performance and skipped classes more often as compared to non-PGs. These findings reiterate that PG has detrimental effects on adolescents’ psychosocial and emotional well being.

Prevalence of adolescent gambling is on the rise (Nower et al. 2004; Turner et al. 2008) and many youths reported gambling at least once at the age of 8–12 years (Ladouceur et al. 1994). Early exposure to gambling may lead to a higher risk of developing PG, which many PGs in retrospect reported gambling at a younger age onset (Chambers et al. 2003; Nower et al. 2004). Furthermore, due to limited developed cognitive ability, adolescents are more susceptible to gambling fallacies (Chambers et al. 2003; Lavoie and Ladouceur 2004); hence, higher PG prevalence compared to adults (Gupta and Derevensky 1998; Nower et al. 2004; Shaffer et al. 1999).

Nevertheless, efforts and actions have been taken to mitigate adverse consequences of gambling in the society, particularly among adolescents. Educational-based prevention programs are excellent grass-roots methods that have been used in several regions for PG prevention (Lavoie and Ladouceur 2004; Turner et al. 2008a, b; Williams and Connolly 2006). Goldston et al. (2008) noted the importance of addressing PG at early age to reduce carryover of PG behaviour into adulthood. Hence, this review aims to examine the available literature on educational-based prevention programs focusing on adolescents. A systematic search was done using keywords of gambling, prevention, awareness, and education as well as inclusion criteria of educational-based approach and adolescent population through the following databases: Academic Search Complete, PsycARTICLES, Google Scholar, Springer, PubMed, Elsevier and ProQuest Central. All papers were further evaluated, and this results in a total of 17 studies, which will be described and evaluated in subsequent sections.

## Educational-based gambling prevention programs among adolescents

Several gambling prevention programs have been developed to address the arising prevalence of PG among adolescents. As prevention outcomes are based on the content and the targeted audience, risk and protective factors related to gambling are essential components in designing preventions. Guilamo-Ramos et al. (2005) noted that preventions can be based on different theoretical perspective, one focusing on addressing unique determinants of behaviours (risk factors) and another focusing on common determinant of behaviours (protective factors). Dowling et al. (2017) defined risk factors as factors that can increase the possibility of PG and protective factors as factors that can reduce the possibility of PG. Hence, prevention programs that target risk factors aim to decrease the influence of risk factors, whereas prevention programs targeting protective factors aim to increase the influence of protective factors in preventing PG. The educational-based gambling prevention programs for adolescents found in this paper are mainly categorized into either the unique or common determinant of problem gambling approach to warrant further discussion. A summary of various prevention programs based on unique and common determinants of problem gambling approach are tabulated in Tables 1 and 2.

**Table 1:** Unique determinant of problem behaviour approach

Authors	Aim and program design	Participants	Findings
Ferland et al. (2002)	Examined a video-based educational program to correct misconceptions and cognitive errors on gambling such as differences between gambling and skill, chances of winning, illusion of control, randomness and winning strategies.	*N* = 424 students in age range from 11 to 15 years old Conditions: (1) video only; (2) information only; (3) information and video; (4) control	A significant reduction of misconceptions, F(3, 416) = 8.56, p < 0.0001, and knowledge errors, F(3, 416) = 8.86, p < 0.0001, reported in all treatment groups compared to the control group Most reduction in misconceptions reported in the information and video condition groups
Ladouceur et al. (2003)	Examined a preventive exercises on correcting gambling misconceptions and made comparison with the Count Me Out gambling awareness program Three exercises: (1) “The Draw” activity focused on illustration of independence of events in gambling and the concept of chance; (2) “The Dice Game” demonstrated that one cannot control the results of gambling; (3) The “Lottery” activity emphasized that no one can control chance	First phase of study, N = 153 students from grade 5 and 6, divided into treatment and control group Second phase of the study, *N* = 509 students from the same grades, conditions: (1) Count me out administered by specialist (C-S); (2) Count me out administered by a teacher (C-T); (3) Preventive exercises designed and administered by specialist (E-S)	In the first study, a significant reduction in erroneous perception reported in the treatment group compared to the control group, F(1, 151) = 13.90; p < .05 In the second study, E-S condition reported significant reduction of misconceptions compared to C-T and C-S conditions, F (2,346) = 3.83; p < .05 Program administered by the specialist produced better outcomes compared to program administered by the teacher (p < .05) Preventive exercises designed by the specialist were found more efficient compared to the exercises from the Count Me Out program (p < .05)
Ladouceur et al. (2004)	Examined an English translated video-based educational program to correct misconceptions and improve knowledge about gambling	*N* = 506 students from grade 7 and 8 divided into treatment and control group	Significant improvement on knowledge and reduction in misconceptions about gambling reported in the treatment group
Lavoie and Ladouceur (2004)	Examined a video-based educational program to modifying attitudes towards gambling and improving knowledge on gambling such as misconceptions, cognitive errors and illusion of control	*N* = 273 French-speaking students in grade 5 and 6 Conditions: (1) Discussion + Video; (2) Video-only; (3) Control	Significant increase in gambling knowledge, *F*(2.266) = 7.25, *p* < .005, and decreases in erroneous attitudes, *F*(2.267) = 7.05, *p* < .005, reported in both treatment groups No significant differences found between the two treatment groups
Ladouceur et al. (2005)	Examined a video-based educational program to improve gambling knowledge and correct gambling misconceptions	*N* = 568 from grade 11–12 from three high schools, divided into treatment and control group	Significant improvement in specific knowledge on excessive gambling, *F*(1,493) = 18.06; *P* < .0001, and decrease stereotypes towards excessive gamblers, *F*(1,481) = 24.36; *P* < .0001, reported in treatment group
Williams and Connolly (2006)	Examined a class educational program to decrease gambling behaviour by improving statistical knowledge such as descriptive statistics, probability, estimation, and the central limit theorem	*N* = 470 university students Conditions: (1) Probability Theory + Gambling Examples; (2) Generic instruction on Probability; (3) Control	Significant increase in statistical knowledge, *F*(2, 330) = 25.4, *p* < .01, and resistance to gambling fallacies, *F*(2, 330) = 3.2, *p* = .05, reported in treatment groups Reported no significant reduction in gambling behaviour
Korn et al. (2006)	Examined the usability internet-based prevention program through games, information and help resources Program includes time management, general risk perception, money management, decision making, principles of randomness, self-assessment, negative consequences minimization, and gambling treatment resources	*N* = 34 youths were interviewed	Youths reported that they liked the information, interface and interactivity of the website, felt that the content were appropriate and appealing, increased knowledge and awareness about gambling, and know where to seek help for gambling related issues
Taylor and Hillyard (2009)	Examined a gambling awareness prevention program “Don’t Gamble Away Our Future” to improve gambling awareness and knowledge	*N* = 8455 students from primary, junior high and high school Parents were invited for presentations and given information packet	Significant change in gambling misconceptions reported in post-test, t (8,454) = − 50.89, p = .000
Walther et al. (2013)	Examined a school-based media education program on gambling knowledge, attitudes and behaviors Program: 90-min program about gambling fallacies, discussion about warning signs of pathological gambling and role play exercise on gambling features	*N* = 2109 students in grade 6 and 7 from 80 schools, divided into treatment and control group	Significant increase in gambling knowledge (d = 0.18), decrease in problematic gambling attitudes (d = 0.15) and current gambling behavior (d = 0.02) No significant effect on lifetime gambling
Todirita and Lupu (2013)	Compared primary prevention program with rational emotive education (REE) on children’s gambling knowledge Primary prevention program focused on illusion of control, attitudes and cognitive errors in gambling REE focused on problem-solving techniques and improving emotional strength	*N* = 81 children of age 12–13 Conditions: (1) Primary Prevention; (2) REE; (3) Control	Significant improvement reported in gambling knowledge in both treatment groups, *F*(2, 77) = 23.33, *p* < .001 Primary prevention was more efficient than REE, *F*(2, 78) = 21.97, *p* < .001, in changing misconceptions about games
Lupu and Lupu (2013)	Compared effectiveness of primary prevention program and REE Primary prevention program focused on restructuring gambling misconceptions REE focused on understanding cognitive and behavior ABC model to gambling	*N* = 75 teenagers from grade 6 Conditions: (1) Control; (2) REE; (3) Primary prevention and REE	Significant change in erroneous cognitions about gambling reported in treatment group that was exposed to primary prevention program and REE, F(2, 72) = 33.54, p = .000, changes maintained and lasted for at least 12 months
Donati et al. (2014)	Examined a school-based educational program to prevent adolescent PG focusing on gambling knowledge and misconceptions, economic gambling perception and superstitious thinking	*N* = 181, mean age = 15.95	Significant improvement in gambling knowledge, F(1,145) = 12.62, p < .01, and economic perception, F(1,143) = 7.16, p < .01, in reported treatment group Significant reduction in gambling misconceptions, F(1,145) = 10.84, p < .01, and superstitious thinking, F(1,141) = 5.48, p < .05 reported in treatment group Findings were stable over time and small significant gambling behavioural change were reported, *χ* ^2^ (1, N = 83) = 2.34, p < .05

**Table 2:** Common determinant of problem behaviour approach

Authors	Design	Participants	Findings
King and Hardy (2006)	Developed gambling action team (GAT) to address campus gambling Prioritize in: (a) developing comprehensive gambling education program; (b) providing gambling related consultation; (c) developing problem gambling awareness approach; (d) ensuring compliance with local efforts, law and legislation	Participants: Students from University of Alabama campus	Effectiveness of the program was not evaluated The program was comprehensive as it targeted: (a) awareness and information; b) skill development and education; c) capacity building, community development and institutional change; (d) social and public policy; prevention aimed at individuals at high risk
Turner et al. (2008a)	Examined a 1 h prevention program for PG, targeting gambling myths, poor coping skills, emotional distress and problem solving skills	*N* = 374 from grade 5 to 12 from 18 schools, divided into treatment and control group	Significant improvements on gambling misconceptions reported, F (1,360) = 6.8, p < 0.01, in the treatment group but no significant effect on gambling behaviour, coping skills and gambling attitudes
Turner et al. (2008a)	Examined a school-based educational program focusing on PG awareness, self-monitoring and coping skills and random chance knowledge	*N* = 201 students of age 15–18, divided into treatment and control group	Significant increased in knowledge in random chances, *F*(1, 9.4) = 14.7, *p* < .01, self-monitoring, *F*(1, 8.4) = 6.4, *p* < .05, and coping skills, *F*(1, 9.6) = 9.7, *p* < .02, reported in treatment group Found a moderate to high impact on students who were at-risks
Williams et al. (2010)	Examined a school-based prevention program to prevent PG Five to six interactive lessons: (1) history of gambling; (2) problem gambling; (3) gambling fallacies; (4) decision making and problem solving, and (5) barriers to good decision making and problem solving	*N* = 1240 students from grade 9–12 from 14 schools, divided into treatment and control group	Significant improvements in gambling knowledge, *F*(2, 1235) = 35.1, *p* < .001), gambling attitudes, *F*(2, 1235) = 15.4, *p* < .001, decision making, problem solving, *F*(2, 1235) = 6.29, *p* = .002, resistance to gambling fallacies *F*(2, 1235) = 34.4, *p* < .001, reported in treatment group. More negative attitudes towards gambling Significant reduction in rates of gambling frequency, *F*(2, 1235) = 4.07, *p* = .017, and PG, *χ* ^*2*^ (1 *df*) = 3.75, *p* = .053, reported in treatment group
Luk et al. (2011)	Examined a positive youth development program (P.A.T.H.S) to help students to develop intrapersonal, interpersonal skills and sense of personal autonomy Focus group conducted to examine the impression and perceived benefits of the program	*N* = 232 secondary one students from two schools, 16 students selected for focus group sessions	Significant positive change in the social competency scores (p = .0001) compared to the pre-test Significant reduction in life satisfaction scale and an increase in behavioral intention to drink alcohol and to gamble Program was reported boring by some students Improvements on interpersonal skills, emotion management and a sense of responsibility reported

## Unique determinant (risk factors) of problem gambling approach

In the gambling prevention context, much emphasis has been placed on addressing unique gambling-related cognition such as erroneous beliefs about gambling (Turner et al. 2008a, b). Gamblers’ faulty beliefs and lack of gambling knowledge contribute to the development of PG (Blaszczynski and Nower 2002), which suggests the importance of educational gambling information in PG prevention. Education of information, based on the inoculation theory, states that knowledge can prepare individuals against future gambling urges (McGuire 1961). In other words, educational gambling knowledge serves as a resistance that can help protect individuals from future attitudinal change.

Mathematical education has been proposed to be effective in correcting erroneous cognitions on gambling (Lavoie and Ladouceur 2004; Turner et al. 2008a, b; Williams and Connolly 2006; Williams et al. 2010). Williams and Connolly (2006) investigated the effectiveness of increasing statistical and mathematical knowledge through classes focusing on gambling probabilities to reduce gambling participation among university students. A post 6 months evaluation then showed that students’ knowledge in gambling odds and resistance towards gambling fallacies increased after the prevention, but no visible changes in actual gambling behaviour were reported. This suggests that mathematical knowledge contributed to the students’ theoretical knowledge may be insufficient to induce changes in PG behaviour. In fact, PGs are found to be well-equipped with gambling knowledge and information (Delfabbro et al. 2009). The differences between PGs and non-PGs could be explained by the unrealistic beliefs they hold about gambling. Goldston et al. (2008) noted that misconceptions about illusion of control can contribute to GD. This belief system seems to reduce their rationality when gambling, focusing on winning strategies instead of the amount of time and money spent in gambling activities.

This leads to prevention efforts focused on addressing gambling misconceptions. Lavoie and Ladouceur (2004) conducted a video-based prevention program to increase students’ gambling knowledge and modify erroneous gambling perceptions by showing a video about illusion of control and gambling probabilities. Video was used as a medium as it could capture students’ interest and attention, resulting in larger prevention effect. Results revealed that students’ gambling knowledge increased and erroneous perceptions were reduced. This implied the benefits and effectiveness of multimedia learning in illustrating complicated gambling-related concepts, which can prevent PG behaviour (Mayer and Moreno 2002; Wohl et al. 2010). It is important to note that the impact of video presentations on actual gambling behaviour was not evaluated in this study, which could be further examined.

Goldston et al. (2008) developed a video-based prevention aimed to educate students about gambling misconceptions. Students aged between 11 and 15 years old were recruited and assigned into either video-only, information-only, both video and information, or control condition. In the video condition, students watched a 20-min video about gambling and skill, chances of winning, randomness, and illusion of control through a humorous presentation portrayed by a character in the video. Meanwhile, students in the information-only condition engaged in discussions about gambling activities, misconceptions, financial consequences and development of lotteries. Results revealed that students in all three conditions reported a significant decrease in gambling misconceptions compared to the control group. The combined information and video condition also showed greater treatment effect compared to the other treatment conditions. Results indicate that humour is an effective way to capture the youth’s attention and to create a fun learning environment. Moreover, interactive sessions that encourage engagement and discussions, as compared to a didactic session is more effective for youths. Holm (2000) agrees that interactive education is more effective in changing performance as compared to didactic education.

In a follow up study by Ladouceur et al. (2004), the same video was translated into English and examined on a group of English-educated students from seventh to eighth grade. Similarly, students who watched the video had a significant improvement on gambling knowledge and a reduction in gambling misconceptions compared to the control group. This lends support for the usage of video to educate youth about gambling and correct their misconceptions about gambling. However, this study did not compare between the treatment conditions, so the conclusions on which method of delivery was better could not be determined. Ladouceur et al. (2005) conducted a similar study and found similar results in the treatment group. Nonetheless, research findings generally supported the benefits of video presentation and humor in gambling awareness programs in targeting young audiences.

Apart from these, Ladouceur et al. (2003) conducted a study to examine the effectiveness of a prevention program designed by a psychologist specialized in PG to correct gambling misconceptions through education on the concept of chance and independence of events in gambling outcomes. The program was later compared with another generalist-developed gambling awareness program (Count Me Out), which addressed the concepts of chance, luck and competency in hopes of preventing gambling decisions made based on chance or superstition. The prevention program designed and administered by a specialist was more effective in reducing gambling misconceptions, which suggested the significance of both program administrator and content specificity. Donati et al. (2014) also examined an educational program, conducted by trained developmental psychologists, which targeted gambling misconceptions, economic gambling perception and superstitions. Positive outcomes were stable over time and some gambling behavioral changes were reported. These findings highlighted the importance of training prevention program providers and developing programs with solid theoretical foundation.

Walther et al. (2013) examined the short-term effects of a media education prevention for sixth and seventh grade students on their gambling knowledge, attitudes and behaviours. The whole program included internet use, online communication, online gaming and gambling. The 90-min program focused on educating students about gambling fallacies, signs of pathological gambling and gambling features. Students in the treatment group reported an increase in gambling knowledge, and reduction in both problematic gambling attitudes and current gambling behaviour seven weeks after intervention. However, there was no significant effect on lifetime gamblers. The results indicate that long-term effects should be taken into consideration when analyzing the effectiveness of gambling prevention programs on adolescent by examining the effects during adulthood. Long-term effect is important in prevention studies because individuals interact with developmental, societal and cultural factors that constantly affect cognition and behavior, consequently impacting on the potential escalation (or non-escalation) to PG. Prevention programs should have a long-term goal to assist adolescents in coping with gambling urges while maintaining a healthy lifestyle. Furthermore, media education can potentially assist students in developing critical analytical skills necessary in processing positive media portrayals of gambling wins, which consequently reduces gambling propensity for leisure purposes.

In another study, Korn et al. (2006) developed a website to educate youths about gambling activities through multiple prevention strategies which targeted a range of gambling behaviours. Participants engaged in interactive games and learned about time and money management, general risk perception, decision making, and concept of randomness. Self-assessment and negative consequences minimization (i.e., for identified high-risk PGs) were provided to assist participants in assessing their PG severity. Treatment resources were made available to participants who need professional help in managing their gambling behaviour. Finally, participants were interviewed to assess the effectiveness and impression of the website. Results showed that participants liked the presentation and interaction of the website, felt that the website was user friendly, content appealing and appropriate, as well as, managed to gain knowledge and awareness about gambling. Future studies building up on these findings will benefit from utilizing the internet as a medium for PG awareness programs among youths, by introducing an interactive website as an effective means to retain interest and convey information to younger individuals. Proudfoot et al. (2011) stated that internet intervention is ideal as it can be tailor made to meet the needs of individuals with differing levels of PG severity. Furthermore, this study highlighted the importance of strengthening the social support system in improving youths’ coping strategies and managing risk factors.

Focusing on the social support aspect, Taylor and Hillyard (2009) examined an interactive prevention program to raise gambling awareness among students, school administrators and parents. Students participated in lectures, discussions and activities to understand gambling and dangers associated to it, while parents were invited to presentations and given an information packet. Significant improvements in gambling knowledge and awareness were reported after the program. Although the inclusion of parents as social support for participating youths were positively accepted, outcome effects of parents’ participation was not empirically examined to determine the extent of positive impact on students’ outcomes.

Besides using media as a medium to deliver preventions, some studies examine other prevention approach and compared the effectiveness of the programs. A recent work by Todirita and Lupu (2013) compared one program using specific information about gambling and the other using rational emotive education (REE). REE targets to enhance emotions strength through increase awareness of emotional distress caused by irrational beliefs in gambling, which allows irrational beliefs to be replaced by rational beliefs. Lupu and Iftene (2009) found that REE can reduce anxiety associated with some disruptive behaviours such as gambling. Students in the information group were given hands-on experience on gambling activities via an interactive software, whereas students in REE group were taught skills on identifying emotions using the Activating-Belief-Consequences (ABC) model which explained that cognitions can triggered negative emotions and disruptive behaviours. Through this model, students learnt to change their feelings and behaviours through altering erroneous cognitions. Students in both groups reported improvements in gambling knowledge, particularly those in the information group who reported significant improvements in knowledge of gambling, illusion of control and erroneous cognitions (Todirita and Lupu 2013). The greater impact in information group could be moderated by age, as the students recruited were between 12 and 13 years old. According to Piaget’s cognitive development theory, children are still in a concrete operation stage, which they have limited ability in solving concrete problems (Derevensky et al. 1996). The REE technique might be a difficult concept to grasp; hence, pure information via interactive software might be more appropriate for their age. Future research can examine how different prevention approach can affect different age group. Lupu and Lupu (2013) replicated a similar study by comparing the effectiveness of program which included both information and REE approaches, results showed that a combination of both approaches yielded larger intervention effects that were long-lasting (12 months). This opens up the possibility of including multiple approaches that targets both cognitive and emotions aspect for better and longer-lasting outcomes.

The effectiveness of school-based prevention programs based on unique determinant of behaviour has found to show positive results on increasing gambling knowledge and correcting gambling misconceptions. However, the impact on actual gambling behaviour was not well-established. The unique determinant approach also mainly focused on cognitive factors, less emphasis was placed on other factors such as emotions, culture, societal and family influence. Blaszczynski and Nower (2002) found that emotional vulnerabilities and prior impulsive-related disorder contributed to the development of problem gambling as well. Hodgins et al. (2012) also found that PG behaviour was associated with religion, demographic variables, peers’ influence and marital status. Hence, preventions that are based on specific determinant of gambling behaviour should examine these factors as well. On the other hand, an alternative view, which focuses not on problem behaviours, but emphasizes on positive factors in adolescent development associated with problematic behaviours can provide valuable insights. This view is based on common determinant of behaviours and is based on the assumption that positive development in youth can act as a protective factor against problematic behaviours (Guilamo-Ramos et al. 2005). Preventions developed based on this concept emphasize on building social skills, coping skills, resilience, self-efficacy, spirituality and any other positive behaviours that can potentially reduce or prevent negative behaviours.

## Common determinants (protective factors) of problem gambling approach

Prevention studies that adopt the common determinant approach utilize the positive psychology approach, which is to focus on enhancing the protective factors to reduce the influence of risk factors that contribute towards PG. In gambling prevention studies, only a few studies were found using the common determinant approach as strategy in prevention programs. Turner et al. (2008a, b) designed a curriculum to teach gambling probabilities, self-monitoring, and coping skills. The implemented curriculum lasted seven weeks with teachers carrying out various interactive activities such as discussion, skits and counseling sessions. The findings revealed that the experimental group showed significant improvements in their understanding on randomness, self-monitoring, and coping skills. This program had greater impact especially for those students who needed such information; however, not for those high-risk students in terms of coping skill knowledge (Turner et al. 2008a, b).

Similarly, Turner et al. (2008b) examined the effectiveness of a 1-h program targeting gambling myths, poor coping skills, emotional distress and problem solving skills on students of grade five to 12. Researchers reported significant improvements on gambling misconceptions, but there were no significant results reported for coping skills, gambling attitudes and behaviour. One possibility of such outcome could be due to the short-term exposure to the program, which was insufficient to create a meaningful impact on skills, attitudes and behaviour. The program only successfully changed the cognitive aspect due to the informative element. This highlights the importance of the duration of exposure to programs in order for internalization to occur, which in turn facilitates change in attitude and behaviour.

Williams et al. (2010) conducted a 4 months (longer exposure) program on lessons regarding gambling knowledge, erroneous cognitions and coping skills. The program mainly covered five lessons on gambling history, PG, gambling fallacies, decision making, and problems solving skills. Besides reporting increased gambling related knowledge, students gained better resistance to erroneous cognitions, and improved problem solving abilities and decision-making skills. Furthermore, participants had a more negative attitude towards gambling and showed a decline in PG frequency. The results for both Turner et al. (2008a, b) and Williams et al. (2010) suggested that domains like coping skill and problem solving can be effective in addressing PG.

Although no apparent evaluation was conducted to measure program effectiveness, the work of King and Hardy (2006) is worth a mention for the comprehensive coverage in a college setting. The main core focus in the program was the formation of a team known as the Gambling Action Team (GAT) that takes specific initiatives in gambling education using proactive means. The GAT focused on developing a comprehensive gambling education, gambling-related consultation, PG awareness, and ensuring compliance with governmental efforts and legislation. This comprehensive program targets the collaboration of many experts and advocates to prevent PG using multiple platforms, such as gambling symposiums, gambling and debt management counseling based on campus, advertisements and informational websites (King and Hardy 2006). Although evaluation of its effectiveness is yet to be conducted, the GAT preparation and implementation was said to be a comprehensive program as it covers various important aspects such as awareness, skill development and capacity building (Connecticut Council on Problem Gambling 1998).

In Asia, limited studies are available on gambling prevention among youths. Luk et al. (2011) looked into the impact of a positive youth development program on gambling behaviours of secondary students in Macau. The program aimed to improve interpersonal, intrapersonal skills, and sense of autonomy to prevent problem behaviours such as alcohol abuse and gambling. Outcomes showed that there was a significant improvement on social competency scores, but students reported a reduction in life satisfaction and increased behavioral intention to drink alcohol and gamble. Focus group discussions revealed that some students felt that the program was boring. Another possible explanation could be that the interpersonal skills training contributed to increase peer influence to gamble due to socialization. This denotes the importance of emphasizing youth training on coping strategies in dealing with peer pressure to gamble.

The effectiveness of common determinant of behavior approach in prevention effort has not been well established as there were only few studies that found the use of this approach in the literature. Nonetheless, these studies provide some insights to mitigate PG from another perspective. Compared to prevention targeting specific risk factors of gambling, a broader and multidimensional approach might be good to address PG in a more holistic manner by including developmental and environmental factors. It is challenging, however, to select relevant components for program inclusion and determine outcome measures of program effectiveness. Stice and Shaw (2004) noted that many moderating factors can affect intervention outcomes such as participants’ condition, demographic variables, program format, content, number of sessions and measures used. Future research should look into these factors as part of program evaluation to effectively address PG in the community.

## Discussion

Educational-based programs that adopted the unique determinant approach, which targeted risk factors to prevent PG among adolescents, have shown consistent program effect in increasing knowledge and correcting misconceptions about gambling, and consequently increase resistance towards gambling myths and fallacies. However, there is insufficient evidence from these programs to conclude that having good gambling knowledge and belief system can effectively reduce actual youth gambling behaviour. This implies that there is a lack of transference of knowledge and beliefs learnt towards behavioural change in gambling. Future studies can examine programs that use cognitive-behavioural approach to provide opportunities for knowledge application into gambling settings. Another reason for the lack of evidence is due to limited research found that examined behavioural outcomes of the educational programs, this suggests the importance of doing more follow-up studies to examine the effectiveness of programs on gambling behaviour.

Gambling educational programs that targeted risk factors provide useful insights into improving and restructuring cognitive process in relation to gambling. Most studies discussed placed too much emphasis on cognitive aspects that can contribute to gambling behaviour, while other risk factors such as parental and peer influences, which are known contributing factors to youth PG development were not addressed (Vachon et al. 2004; Magoon and Ingersoll 2006; Barnes et al. 1999). As adolescent learn through modelling and are in the stage where they are more susceptible to peer influences and peer pressure, programs that integrate parental and peer support can provide a holistic prevention against PG

On the other hand, programs that adopt the common determinant approach aim to increase the influence of protective factors to address PG among youths provide an alternative perspective. Williams et al. (2010) found that addressing gambling knowledge and fallacies, as well as increasing problem solving skills could reduce gambling behaviour. This suggests the importance of focusing on potential protective factors that can help to address PG. Shek and Lee (2010) suggest that programs that focus on positive youth development has the potential to strengthen their positive qualities and reduce the probability of developing PG. There is also a need to investigate and develop more theories that support this approach, so that more programs can apply appropriate theories into the framework to enhance the effectiveness of the programs. Williams et al. (2010) highlight the importance of incorporating both unique and common determinant approaches into the prevention program due to the benefits of both approaches. Therefore, future research can tap more onto these two approaches, to create more opportunities for both approaches to integrate and complement one another in the effort to mitigate PG among adolescent.

Examining approaches used in different prevention programs provide valuable insights toward building a strong foundation for future programs to be based on. Apart from looking at the approaches, some other factors are important to be discussed in this paper as well. Studies by Korn et al. (2006) demonstrated that youth responded well in programs that were interactive, fun and engaging. Some studies noted the benefits of using multi-media learning to enhance the learning and retention of knowledge among youths [Ferland et al. (2002); Ladouceur et al. (2004); Lavoie and Ladouceur (2004)]. Programs conducted by trained specialist produced better outcomes compared to program conducted by untrained teachers (Todirita and Lupu 2013). These studies point towards the importance of examining other components of programs such as method of delivery as well as the person delivering the programs. A comparison studies by Turner et al. (2008b) and Turner et al. (2008a) that targeted similar skills set in the programs but differed in duration of program showed that the time or number of sessions conducted could have different effects on program outcomes. Stice and Shaw (2004) suggest that different components of programs such as delivery method, content and provider could influence participants’ acceptance and respond towards the programs. This opens up more possibility to investigate these factors to enhance the effectiveness of current and future programs.

Given the importance of examining content (approaches, other potential risk and protective factors) and components (delivery method, deliverer, duration and number of sessions) in educational programs, the evaluation of programs should be emphasized as well. Follow up evaluations on actual PG behaviour should be conducted to investigate long-term effects of prevention programs, as youths who are exposed to gambling at a young age could potentially develop PG during adulthood (Kourgiantakis et al. 2016). This indicated that PG can develop throughout youths’ developmental life span, highlighting the need for programs with sustained long-term effect on actual PG outcomes and mental health.

## Conclusion

In summary, the current review paper outlined studies that focus on educational-based gambling prevention programs for adolescents. Emerging discussions here emphasize the need for more theoretical and evidence-based programs that examine approaches, potential risk and protective factors, program structure, delivery methods and structured long-term evaluation. All these factors should be taken into consideration by future researchers in developing and implementing programs that can effectively mitigate PG among adolescent.
